# Effects of *in Utero* Exposure to Arsenic during the Second Half of Gestation on Reproductive End Points and Metabolic Parameters in Female CD-1 Mice

**DOI:** 10.1289/ehp.1509703

**Published:** 2015-08-21

**Authors:** Karina F. Rodriguez, Erica K. Ungewitter, Yasmin Crespo-Mejias, Chang Liu, Barbara Nicol, Grace E. Kissling, Humphrey Hung-Chang Yao

**Affiliations:** 1 Reproductive Developmental Biology Group, Reproductive and Developmental Biology Laboratory, and; 2 Biostatistics Branch, National Toxicology Program, National Institute of Environmental Health Sciences, National Institutes of Health, Department of Health and Human Services, Research Triangle Park, North Carolina, USA

## Abstract

**Background:**

Mice exposed to high levels of arsenic *in utero* have increased susceptibility to tumors such as hepatic and pulmonary carcinomas when they reach adulthood. However, the effects of *in utero* arsenic exposure on general physiological functions such as reproduction and metabolism remain unclear.

**Objectives:**

We evaluated the effects of *in utero* exposure to inorganic arsenic at the U.S. Environmental Protection Agency (EPA) drinking water standard (10 ppb) and at tumor-inducing levels (42.5 ppm) on reproductive end points and metabolic parameters when the exposed females reached adulthood.

**Methods:**

Pregnant CD-1 mice were exposed to sodium arsenite [none (control), 10 ppb, or 42.5 ppm] in drinking water from gestational day 10 to birth, the window of organ formation. At birth, exposed offspring were fostered to unexposed dams. We examined reproductive end points (age at vaginal opening, reproductive hormone levels, estrous cyclicity, and fertility) and metabolic parameters (body weight changes, hormone levels, body fat content, and glucose tolerance) in the exposed females when they reached adulthood.

**Results:**

Arsenic-exposed females (10 ppb and 42.5 ppm) exhibited early onset of vaginal opening. Fertility was not affected when females were exposed to the 10-ppb dose. However, the number of litters per female was decreased in females exposed to 42.5 ppm of arsenic *in utero*. In both 10-ppb and 42.5-ppm groups, arsenic-exposed females had significantly greater body weight gain, body fat content, and glucose intolerance.

**Conclusion:**

Our findings revealed unexpected effects of *in utero* exposure to arsenic: exposure to both a human-relevant low dose and a tumor-inducing level led to early onset of vaginal opening and to obesity in female CD-1 mice.

**Citation:**

Rodriguez KF, Ungewitter EK, Crespo-Mejias Y, Liu C, Nicol B, Kissling GE, Yao HH. 2016. Effects of *in utero* exposure to arsenic during the second half of gestation on reproductive end points and metabolic parameters in female CD-1 mice. Environ Health Perspect 124:336–343; http://dx.doi.org/10.1289/ehp.1509703

## Introduction

Developmental origins of adult disease are implicated in cardiovascular diseases (Barker et al. 1989; Forsén et al. 1999), diabetes (Fall et al. 1998; Vignini et al. 2012), cancers, and reproductive disorders such as polycystic ovarian syndrome (Xita and Tsatsoulis 2010; Xu et al. 2014). The nutritional and physical status of the mothers and their exposure to various environmental toxicants during pregnancy are contributing factors to their offspring’s susceptibility to various diseases when they reach adulthood (reviewed by Boekelheide et al. 2012). One such environmental toxicant is inorganic arsenic. Arsenic is a metalloid that is naturally found in the environment and is a common contaminant in drinking water (Smedley and Kinniburgh 2002) and in crops such as rice (Charnley 2014). In the United States, the maximum contaminant level of arsenic in the drinking water set by the U.S. Environmental Protection Agency (EPA) is 10 ppb (parts per billion; U.S. EPA 2002). Many private wells in the United States and groundwater in other parts of the world have arsenic levels > 10 ppb (even > 5,000 ppb; reviewed by Smedley and Kinniburgh 2002).

Gestation is a sensitive period for arsenic toxicity (Devesa et al. 2006; Kozul-Horvath et al. 2012). In humans, chronic exposure to inorganic arsenic has been linked to cardiovascular disease, diabetes mellitus, and cancers of the skin, lung, liver, urinary bladder, and prostate (Bräuner et al. 2014; Moon et al. 2013; Smith et al. 2013; Steinmaus et al. 2014). In mice, *in utero* exposure to arsenic (in doses ranging from 42.5 to 85 ppm) resulted in an increased incidence of lung, liver, adrenal, skin, and ovarian tumors when the exposed embryos reached adulthood (Liu et al. 2007; Tokar et al. 2010; Waalkes et al. 2004b). The urogenital system is a known target for arsenic toxicity: CD-1 mice exposed to 85 ppm arsenic *in utero* from embryonic day 8 (E8) to E18 exhibited increased incidence of ovarian, uterine, and adrenal gland tumors at 90 weeks of age (Waalkes et al. 2006).

Although the detrimental impacts of high-level arsenic exposure (in the parts per million range) are well documented, it is not clear what the consequences of exposure to arsenic at levels relevant to normal human consumption (in the parts per billion range) may be. Exposure to 50 ppb arsenic from fetal life to adulthood increased lung tumor incidence in female CD-1 mice (Waalkes et al. 2014), and exposure to 10 ppb arsenic during pregnancy resulted in liver steatosis and decreased breast milk triglyceride levels in exposed C57BL6/J dams, leading to growth deficits in their offspring (Kozul-Horvath et al. 2012). In the present study, we investigated the effects of 10 ppb arsenic [the maximum contaminant level (MCL) in drinking water designated by the U.S. EPA (2001)] for the relevance of this level to human exposure. We also exposed mice to 42.5 ppm arsenic in drinking water to define the impact of *in utero* arsenic exposure at a known tumor-inducing level (Tokar et al. 2010; Waalkes et al. 2003) on general physiological functions from puberty to 1 year of age. The exposure period was restricted to the second half of gestation from E10 to birth (the critical window of fetal organ formation in mice), and the animals were allowed to develop to adulthood without further exposure. We focused on reproductive and metabolic endpoints, which are known to have physiological interactions.

## Materials and Methods

**Figure 1:**
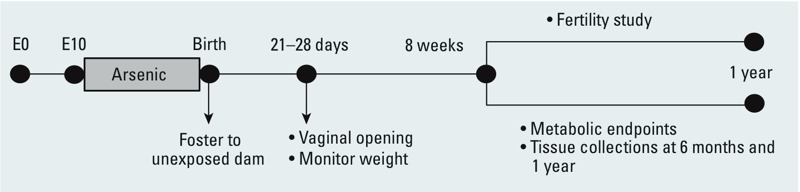
Experimental design: Pregnant CD-1 females were exposed to 0 (control), 10 ppb, or 42.5 ppm sodium arsenite in drinking water from E10 to birth. At birth, pups were fostered to females that had not been not exposed to arsenic. Vaginal opening was checked daily starting at 18 days of age, and body weight was recorded weekly for 15 weeks. At 8 weeks of age, female pups were included in the fertility study or analyzed for metabolic end points. The sample size for each experiment is listed in Table 1.

*Animals and treatments.* Female CD-1 mice, 8–10 weeks old (Charles River, Wilmington, MA), were timed-mated with CD-1 males. The day that the vaginal plug was detected was considered embryonic day 0 (E0), and the pregnant females were housed individually in plastic cages using Sani-Chips® bedding (P.J. Murphy Forest Products Corp.). The pregnant females were provided *ad libitum* with NIH-31 chow (Harlan Teklad, Indianapolis, IN) and water processed through a reverse-osmosis deionized system (Hydro Service and Supplies, Inc., Durham, NC). Arsenic was below detectable levels in the NIH-31 chow (analyzed by inductively coupled plasma atomic emission spectroscopy; Microbac Laboratories). At E10, pregnant females were randomly assigned to one of the following treatment groups (11 pregnant females per group): *a*) control, no inorganic arsenic; *b*) 10 ppb inorganic arsenic (as sodium arsenite; Spectrum Chemicals, New Brunswick, NJ); or *c*) 42.5 ppm inorganic arsenic. Arsenic was administered in the drinking water. The treatment window was from E10 to birth. Pregnant females were allowed to deliver naturally, and newborn pups were immediately fostered to females that had not been exposed to arsenic. In order to ensure even growth of the pups, each foster female was given 10 newborns from the same litter. Female pups from each litter were assigned to the experiments listed in Table 1. The timeline of the experiments is outlined in Figure 1. All animals were maintained in standard plastic mouse cages (maximum of 5 mice per cage) in temperature-controlled rooms and under controlled lighting (12 hr light:12 hr dark). Euthanasia was performed by CO_2_ inhalation. All animal procedures were approved by the National Institutes of Health Animal Care and Use Committee and were performed in accordance with an approved National Institute of Environmental Health Sciences animal study proposal no. 2012-0006. All animals were treated humanely with regard to alleviation of suffering and followed the *Guide for the Care and Use of Laboratory Animals* (Institute of Laboratory Animal Resources 2011).

**Table 1:** Figure assignment, end points of measurement, and sample sizes.

Figure	End points	Sample size
Figure 2	Vaginal opening from 18 to 29 days of age. Body weight to 8 weeks of age	Control: 29 10 ppb: 37 42.5 ppm: 35
Figure 3	LH, FSH, and estradiol	Control: 5–6 10 ppb: 5–6 42.5 ppm: 4–8
Figure 4A	Body weight from 9 to 15 weeks of age	Control: 17 10 ppb: 25 42.5 ppm: 21
Figure 4B	Body fat composition at 4.5 months of age	Control: 9 10 ppb: 10 42.5 ppm: 14
Figure 4C	Glucose tolerance at 5 months of age	Control: 7 10 ppb: 10 42.5 ppm: 11
Figure 5	Body weight and levels of leptin and insulin at 6 months of age	Control: 5 10 ppb: 7 42.5 ppm: 6
Table 2	Estrous cyclicity for 18 days starting at 10 weeks of age	Control: 17 10 ppb: 22 42.5 ppm: 21
Table 3	Fertility: number of litters, total number of pups per litter, total number of pups per female, days between litters, and fertile period	Control: 5 10 ppb: 8 42.5 ppm: 13
Sup Figure S1A,B	Maternal weight gain and litter size	Control: 7 10 ppb: 7 42.5 ppm: 7
Sup Figure S1C	Fetal body weight at E18	Control: 46 10 ppb: 30 42.5 ppm: 38
Sup Figure S2	Ovarian histology at 21 days, 28 days, and 6 months of age	Control: 5 10 ppb: 7 42.5 ppm: 6
Sup Figure S3	Serum levels of estradiol, testosterone, progesterone, and DHEA at 6 months and 1 year of age	Control: 5 10 ppb: 7 42.5 ppm: 6
DHEA, dehydroepiandrosterone; E18, embryonic day 18; FSH, follicle-stimulating hormone; LH, luteinizing hormone; Sup, Supplemental Material.		

*Vaginal opening and estrous cyclicity analysis.* Female pups (*n* = 29 for the control; *n* = 37 for the 10-ppb group; *n* = 35 for the 42.5-ppm group) were checked at 0900 hours daily for status of the vaginal opening starting at 18 days of age until the day that each female exhibited an open vaginal canal. Estrous cycle was monitored in females starting at 10 weeks of age by vaginal smears taken daily (0900 hours) in the cage for 18 consecutive days. Vaginal smears were immediately fixed on glass slides (Safetex; Andwin Scientific) and stained with hematoxylin and eosin (H&E) following standard H&E protocols. Phases of the estrous cycle were determined based on vaginal cytology as previously described (Jayes et al. 2014).

*Fertility study.* When females reached 8 weeks of age, each was placed in a continuing mating scheme. Breeding pairs of females (control: *n* = 5; 10 ppb: *n* = 8; 42.5 ppm: *n* = 13) and proven males that had not been exposed to arsenic (CD-1 males, 10–12 weeks old) were housed together (one pair per cage) until they reached 1 year of age. The parameters analyzed during the 1-year period included days to first litter, days between litters, average number of pups per litter, total number of pups produced per female, total number of litters per female, and fertile period (measured as the number of days from initial mating to the last litter).

*Measurement of body weight and body fat composition.* Mice were weighed weekly starting at weaning (21 days) until 15 weeks of age. Weights of the mice in the fertility study were included only prior to the beginning of the breeding period. The sample sizes for body weight measurement up to 8 weeks of age were *n* = 29 for the control, *n* = 37 for the 10-ppb group, and *n* = 35 for the 42.5-ppm group. The sample sizes for the body weight analysis from 8 to 15 weeks of age were: *n* = 17 for the control, *n* = 25 for the 10-ppb group, and *n* = 21 for the 42.5-ppm group. Body fat composition was analyzed at 4.5 months of age in mice not included in the fertility study (*n* = 9 for the control, *n* = 10 for the 10-ppb group; *n* = 11 for the 42.5-ppm group) using a PIXImus® densitometer (GE Lunar Corporation, Waukesha, WI).

*Serum analysis.* Hormone analysis was performed in serum collected at different time points from females that were not included in the fertility study. Following euthanasia, blood was collected by either cardiac puncture or from the descending vena cava. Serum (collected from non-fasted females) was separated using BD Microtainer™ plastic capillary blood collectors (BD Diagnostics, Franklin Lakes, NJ) and frozen at –80°C. Serum from 21- and 28-day-old females was used to measure levels of luteinizing hormone (LH) and follicle-stimulating hormone (FSH) with a Milliplex MAP Mouse Pituitary Magnetic Bead Panel (catalog number MPTMAG-49K; Millipore, Billerica, MA; control *n* = 29; 10 ppb *n* = 37; 42.5 ppm *n* = 35). Serum from 6-month-old females was used to measure levels of leptin and insulin with a Mouse Metabolic Kit [catalog number N45124A-1; MSD (Meso Scale Discovery), Gaithersburg, Maryland] according to the manufacturer’s protocols (control *n* = 5; 10 ppb *n* = 7; 42.5 ppm *n* = 6). Serum from 21-day-, 28-day-, 6-month-, and 1-year-old females was used to measure levels of estradiol, dehydroepiandrosterone (DHEA), testosterone, and progesterone with a MULTI-SPOT 96 HB 4-Spot Custom Steroid Hormone Panel (catalog number N45CB-1; MSD). Data are presented in Supplemental Material, Figure S3 (control *n* = 5; 10 ppb *n* = 5; 42.5 ppm = 6). All samples were assayed in duplicate.

*Glucose tolerance test.* Five-month-old females that were not included in the fertility study (control *n* = 7; 10 ppb *n* = 10; 42.5 ppm *n* = 11) were fasted overnight, and their baseline glucose levels in serum were determined using a Nova Max® Plus glucometer (Nova Biomedical, Waltham, MA). The mice were then given an intraperitoneal injection of D-glucose (2 mg/g body weight), and blood samples were collected for glucose measurement at 20, 40, 60, 120, and 180 minutes after the injection.

*Additional end points.* The body weight analysis for the E18 embryos is presented in Supplemental Material, Figure S1 (control *n* = 46; 10 ppb *n* = 30; 42.5 ppm *n* = 38). The ovaries collected from animals at postnatal days 21 and 28 and at 6 months (control *n* = 5–7; 10 ppb *n* = 5–8; 42.5 ppm *n* = 6–8 for each time point) were fixed overnight in paraformaldehyde (PFA) and stained with H&E. Results are shown in Supplemental Material, Figure S2.

*Statistical analysis.* The sample size for each experiment is presented in Table 1. Data on age at vaginal opening were analyzed using log-rank statistics and mixed-model analysis of covariance, adjusting for weaning weight and dam and litter effects. The percentage of time spent in each of the four stages (estrus, metestrus, diestrus, or proestrus) for each of the treatment groups was compared using mixed-effects analysis of variance, with dam as a random effect to take correlations into account. Dunnett’s test was used to compare each treatment group with the control group. Body weight and body fat composition were analyzed using a mixed-model analysis of variance (ANOVA) with dam as a random effect to take littermate correlation into consideration. Hormonal levels and fertility data were compared using ANOVA and Tukey’s multiple comparison tests. *p*-Values < 0.05 were considered statistically significant.

## Results

The goal of this study was to investigate how *in utero* exposure to arsenic in drinking water at the U.S. EPA maximum contaminant level (MCL; 10 ppb) and at tumor-inducing levels (42.5 ppm) (Waalkes et al. 2003, 2004b) affects reproductive and metabolic functions when exposed animals reach adulthood. We restricted the exposure period to the second half of gestation to investigate specifically the impact of arsenic on organ formation. Pregnant CD-1 female mice exposed to arsenic showed no effects of treatment on body weight gain and number of pups born per litter (see Supplemental Material, Figure S1). The weights of the fetuses at E18 were similar among treatment groups with the exception of the 10-ppb group, which showed a significant increase in body weight (see Supplemental Material, Figure S1). The pups born from exposed females appeared healthy and without any signs of stress or malformation, and they were able to develop to adulthood for analyses of reproductive and metabolic endpoints.

**Figure 2:**
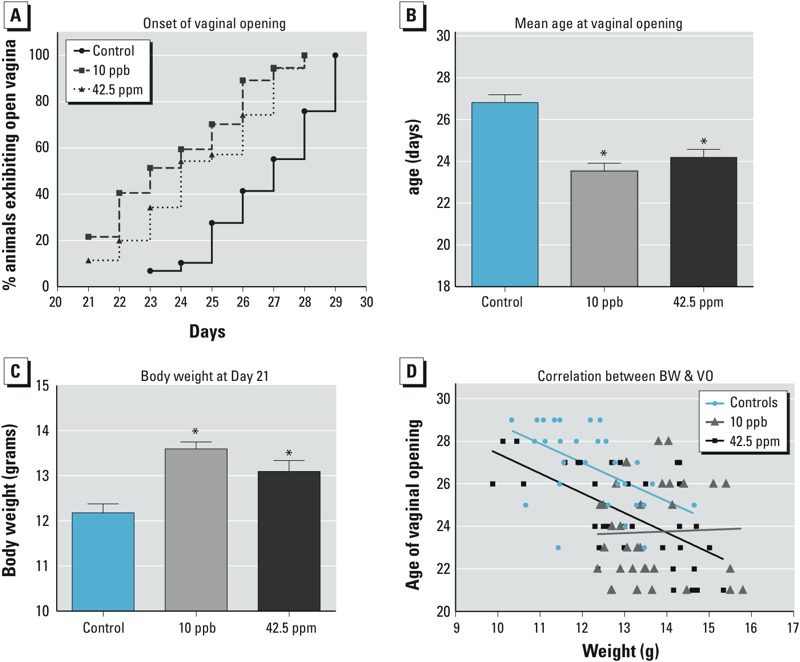
Effects of *in utero* arsenic exposure on onset of vaginal opening and body weight. (*A*) The *x*-axis represents the day of vaginal opening. The *y*-axis represents the percentage of animals with open vaginas. (*B*) The *y*-axis represents the average age ± SE when vaginal opening was observed. (*C*) Average body weight at 21 days of age (average age ± SE); (*D*) lines represent correlations between body weight (BW) and onset of vaginal opening (VO). *p < 0.05.

*Impacts of* in utero *arsenic exposure on reproductive function.* One of the first signs of reproductive development in female mice is the opening of the vagina, an external indicator of the onset of puberty (Hansen et al. 1983). Vaginal opening was first detected in control females at 23 days of age, and by 30 days, all control females exhibited open vaginas (Figure 2A). In contrast, pups exposed to arsenic *in utero* (both 10-ppb and 42.5-ppm groups) exhibited vaginal opening as early as 21 days (Figure 2A). Compared with the control (mean age at vaginal opening, 26.5 ± 0.3 days), the arsenic-exposed females had significantly early onset of vaginal opening (23.8 ± 0.2 days for the 10-ppb group and 24.5 ± 0.3 days for the 42.5-ppm group; Figure 2B). Onset of vaginal opening in mice is known to be positively associated with body weight (Hansen et al. 1983). The pups exposed to either 10 ppb or 42.5 ppm arsenic *in utero* displayed higher body weights at weaning (21 days of age) than controls (*p* < 0.001; Figure 2C). Significant negative correlations between body weight and age at vaginal opening were detected in controls (Figure 2D, *r*^2^ = –0.51; *p* = 0.004) and in the 42.5-ppm group (*r*^2^ = –0.62; *p* < 0.001). However, no significant correlation was observed in the 10-ppb group (*r*^2^ = 0.032; *p* = 0.85), suggesting that exposure to 10 ppb arsenic *in utero* causes early onset of vaginal opening independent of body weight.

**Figure 3:**
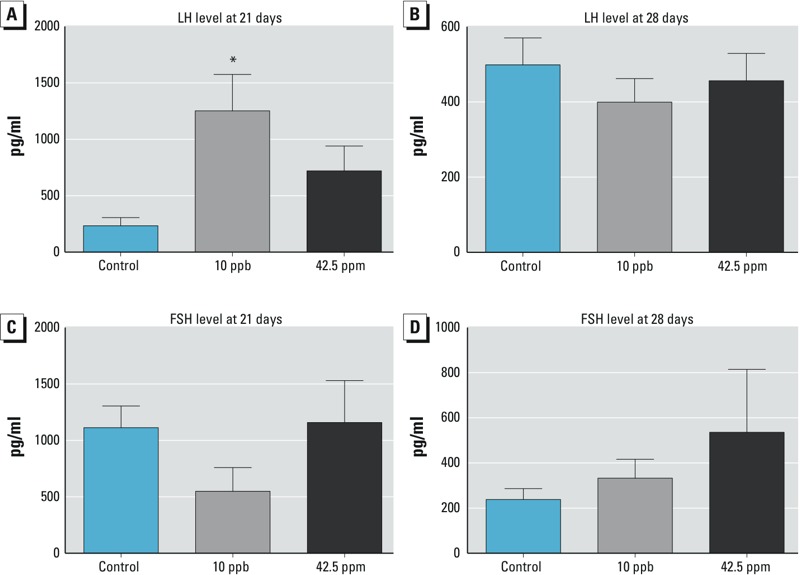
Effects of *in utero* arsenic exposure on levels of serum LH (*A,B*) and FSH (*C,D*) at 21 and 28 days of age (mean ± SE). Abbreviations: LH, luteinizing hormone; FSH, follicle-stimulating hormone; *p < 0.05 compared with control group.

We next examined the levels of gonadotropins (LH and FSH), which are pituitary-derived hormones that trigger reproductive development in females. At 21 days, the serum level of LH was significantly higher in females exposed to 10 ppb arsenic *in utero* than in controls, and this effect was not observed at 28 days (Figure 3A,B); however, no differences were detected at either 21 or 28 days of age in females exposed to 42.5 ppm arsenic *in utero*. Serum FSH level was not significantly different between the control and treatment groups at either time point (Figure 3C,D).

Progression of the estrous cycle is another end point that is indicative of proper reproductive development. We monitored the estrous cycle in exposed females for 18 consecutive days (roughly 3–4 cycles) when they reached 2.5 months of age. The differences in the percentage of time that females spent in each stage of the estrous cycle (P: proestrus, E: estrus, M: metestrus, and D: diestrus) were not statistically significant among the control and treatment groups (Table 2).

**Table 2:** Percentage of time that females spent in each stage of the estrous cycle.

End point	Controls (*n *= 17)	10 ppb (*n *= 22)	42.5 ppm (*n *= 21)	*p*‑Value
Percent in estrus	28.1 ± 2.9	28.8 ± 2.9	27.8 ± 2.2	0.962
Percent in metestrus	31.0 ± 1.4	30.0 ± 2.0	25.9 ± 1.8	0.114
Percent in diestrus	20.6 ± 1.8	24.7 ± 2.2	28.0 ± 2.7	0.092
Percent in proestrus	20.3 ± 1.4	16.4 ± 1.8	18.2 ± 1.5	0.267
Numbers represent averages ± standard error.				

To test whether fertility was affected by *in utero* exposure to arsenic, females were housed with fertile CD-1 males from 8 weeks to 1 year of age, and the fertility outcomes were examined. We found no differences between the control and treatment groups with regard to number of days to first litter, average days between litters, and average number of pups per litter (Table 3). Although no differences were found between the control and treatment groups in the total number of litters born per female, we detected a significant difference between the 10-ppb and 42.5-ppm groups, with fewer litters born, fewer pups per female, a smaller total number of pups born, and a shorter fertile period (measured as the number of days from initial mating to the last litter) for the 42.5-ppm group than for the 10-ppb group (Table 3). No detectable differences were observed in ovarian morphology between control and *in utero*–exposed females at 21 days, 28 days, and 6 months (see Supplemental Material, Figure S2). Although some changes were detected in the pattern of circulating sex steroid levels at 6 months and 1 year (see Supplemental Material, Figure S3), these changes were not statistically significant. In summary, *in utero* exposure to either 10 ppb or 42.5 ppm arsenic resulted in early vaginal opening. Elevated serum LH was also observed in females exposed to 10 ppb arsenic *in utero,* suggesting that these animals may have entered puberty precociously. Despite these reproductive anomalies, the fertility of the exposed females was not significantly different from that of the controls.

**Table 3:** Effects of *in utero* arsenic exposure on the fertility of adult females.

Treatment	Days to 1st litter	Days between litters	Number of pups per litter	Total pups produced per female	Total number of litters per female	Fertile period (days)
Control (*n *= 5)	21.6 ± 0.40	29.04 ± 1.44	13.65 ± 0.93	112.80 ± 12.04	9.00 ± 1.30	254.4 ±18.6
10 ppb (*n *= 8)	22.00 ± 1.62	28.55 ± 1.53	11.03 ± 1.08	124.88 ± 8.56*	10.38 ± 0.94*	302.3 ± 16.5*
42.5 ppm (*n *= 13)	23.31 ± 1.01	31.65 ± 1.59	12.32 ± 0.43	73.77 ± 13.38*	5.77 ± 1.04*	186.1 ± 30.62*
Numbers represent averages ± standard error. *Represents significant differences between 10-ppb and 42.5-ppm groups with *p *< 0.05. There were no significant differences between the treatment groups and the control.						

**Figure 4:**
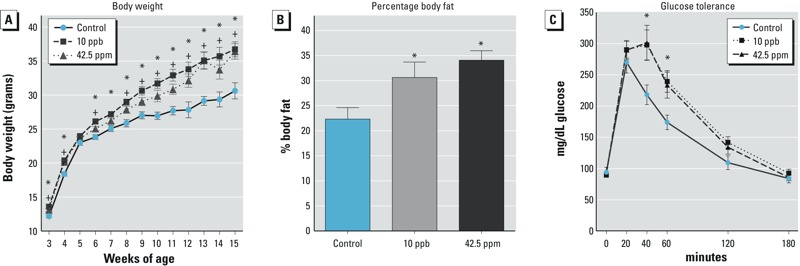
Effects of *in utero* arsenic exposure on (*A*) body weight from 3 to 15 weeks of age. *Indicates significant difference (*p *< 0.05) between the control and the 10-ppb group and + indicates significant difference (*p *< 0.05) between the control and the 42.5-ppm group. (*B*) Percentage body fat. *Indicates significant difference (*p *< 0.05) compared with the control. (*C*) Glucose tolerance analysis. The *y*-axis represents serum glucose levels (mean ± SE; *indicates *p *< 0.05 compared with the control).

*Impacts of* in utero *arsenic exposure on body weight, body composition, and glucose metabolism.* The animals’ increased body weight observed during puberty (Figure 2) prompted us to ask whether this weight increase continued later in life and affected metabolism. We followed body weight changes from 3 to 15 weeks of age in females exposed to arsenic *in utero* (Figure 4A). The body weights of both 10-ppb and 42.5-ppm treatment groups were significantly higher than that of the controls at all time points, particularly after 5 weeks of age. The percentage of fat versus lean mass measured by PIXImus® scans of adult females at 4.5 months was significantly higher in both the 10-ppb (30.6 ± 3%) and 42.5-ppm (34.1 ± 2%) groups than in the controls (22.3 ± 2%; *p* < 0.05) (Figure 4B). In addition to elevated body weight and body fat content, the 10-ppb and 42.5-ppm groups also showed signs of impaired glucose tolerance. Forty minutes after administering a glucose challenge, the serum glucose level in the control group started to decline, whereas the level remained significantly higher in the 10-ppb and 42.5-ppm groups and did not start to decline until the 60-min time point (Figure 4C). It is worth noting that the serum glucose levels before the challenge (time zero) and at 180 min after the challenge were not different among the groups, indicating that the treated animals were not diabetic.

**Figure 5:**
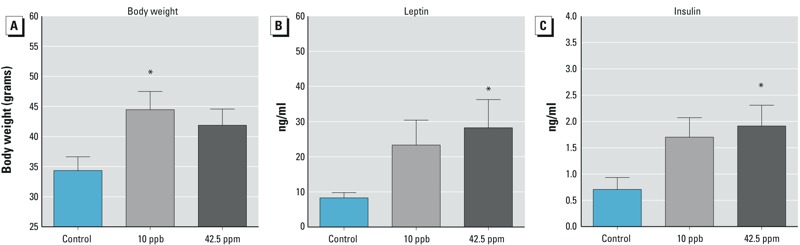
Effects of *in utero* arsenic exposure on (*A*) body weight, (*B*) serum leptin, and (*C*) serum insulin level at 6 months of age (mean ± SE) *p < 0.05 compared with the control.

At 6 months of age, the body weight of females exposed to 10 ppb arsenic *in utero (*44.5 ± 3.0 g) remained higher than that of controls (34.4 ± 2.3 g; *p* < 0.05) (Figure 5A). Serum levels of leptin and insulin, two hormones associated with metabolic syndrome and obesity (reviewed by Fellmann et al. 2013), showed a tendency to be higher in the 10-ppb group than in the controls (Figure 5B,C; *p* = 0.11 for leptin and *p* = 0.06 for insulin) and were statistically significantly different between the controls and the 42.5-ppm group (Figure 5B,C; *p* = 0.05 for leptin and *p* = 0.03 for insulin). In summary, females exposed to 10 ppb and 42.5 ppm arsenic *in utero* became obese starting in young adulthood, likely because of an increase in body fat deposition. Furthermore, exposed females exhibited glucose intolerance, whereas the controls did not. At 6 months of age, the increased body weights of the exposed females remained apparent, and the mice showed tendencies toward elevated levels of circulating leptin and insulin.

## Discussion

Most animal studies on the health impact of arsenic exposures have focused primarily on high doses (ppm level) and on exposure in adulthood. Analyses of arsenic exposure during fetal life indicate that the exposed mice are more susceptible to tumors than controls (Tokar et al. 2010; Waalkes et al. 2004a, 2006, 2007). However, the effects of exposure on other physiological functions remain unclear. In our study, we investigated the impact of *in utero* exposure to U.S. EPA drinking-water standard (10 ppb) and tumor-inducing (42.5 ppm) levels of arsenic on reproductive and metabolic functions when the exposed females reached adulthood. We focused specifically on the window of organ formation (E10 to birth). Unexpectedly, *in utero* exposure to both 10 ppb and 42.5 ppm arsenic resulted in early vaginal opening, an indicator of puberty, and increased body weight compared with that of controls. The pattern of increase in body weight and fat composition is similar to the changes observed in the high-fat diet–induced obesity model in CD-1 female adult mice (Gao et al. 2015; Lei et al. 2007). Therefore, we considered the exposed females in our study to be obese. Although the obesity-inducing high-fat diet was introduced in adulthood, in our study, the potential obesity-inducing agent (arsenic) was only given during fetal life. These observations, along with the tendency for increases in circulating levels of leptin and insulin, suggest that exposure to arsenic during the developmental window from E10 to birth *in utero,* even at levels as low as 10 ppb, could be a contributing factor for obesity and metabolic syndrome in adult female mice.

To our knowledge, the effects of *in utero* exposure to low levels of arsenic on age of vaginal opening in mice have not been reported. However, arsenic exposure, particularly at the parts per million level, has been linked to delayed puberty in other species. In rats, exposure to 10 mg/kg of arsenic in drinking water from 12 days of age to puberty led to delayed sexual maturity (Reilly et al. 2014). Delayed puberty was also observed in rats exposed to 3 ppm arsenic *in utero* until 4 months of age (dams were exposed to arsenic prior to breeding and throughout gestation) (Dávila-Esqueda et al. 2012). These observations suggest that exposure to high levels of arsenic during the peripubertal period may delay reproductive maturity or onset of puberty. Differences in dosing level, length of exposure, age at exposure, and species could contribute to the opposing outcome in our study.

Although early onset of vaginal opening was observed in both the 10-ppb and 42.5-ppm groups, the mechanism underlying this phenotype appears to be different for each group. The negative correlation between body weight and age at vaginal opening was maintained in both the control and 42.5-ppm groups as expected. However, the correlation between body weight and onset of vaginal opening was not observed in females exposed to 10 ppb arsenic. The lack of correlation between body weight and onset of vaginal opening in the 10-ppb group indicates that increased body weight likely does not contribute to precocious puberty at this exposure level. We speculate that our *in utero* exposure paradigm could have a specific effect on the development of the hypothalamic–pituitary–gonadal axis. Age of vaginal opening and levels of circulating LH have been used as indicators of puberty in mice (Risma et al. 1997). Elevated serum LH, which is positively linked to precocious puberty (Risma et al. 1997), was observed only in the 10-ppb-exposed females. In addition to LH, ovary-derived estrogen is involved in onset of vaginal opening. During the prepubertal period, estrogen levels vary from day to day, with a significant decrease on the day of vaginal opening and an increase 2–3 days afterwards (Safranski et al. 1993). We measured the serum estradiol level at 21 and 28 days of age (regardless of the status of vaginal opening) and found that the majority of animals had undetectable levels of estradiol. Therefore, we were not able to establish a connection between estrogen level and early onset of vaginal opening. Although we were unable to detect differences in fertility between the control and treatment groups under our experimental conditions, we observed that females in the 42.5-ppm group had fewer litters, fewer pups per litter, and a shorter fertile period. These data suggest that exposure to 42.5 ppm arsenic has a more dramatic negative effect on fertility than exposure to 10-ppb arsenic. More studies are needed to better understand this phenotype.

The arsenic-induced weight gain was not restricted to prepubertal mice in our study. The significant weight increase continued in adulthood, accompanied by higher body fat content in both 10-ppb and 42.5-ppm groups than in controls. A contributing factor to obesity in adulthood is low birth weight (Beauchamp et al. 2015). To determine whether the weight increase in exposed females was the result of low birth weight, we measured the body weights of exposed embryos at E18, 1 day before birth. We found that following exposure to 10 ppb arsenic, the body weight of E18 fetuses was actually higher than that of the controls, and no differences were detected between controls and embryos in the 42.5-ppm group (see Supplemental Material, Figure S1C). These data suggest that the obesity observed in the exposed mice was not related to low birth weight. A study that investigated the effects of a single exposure of arsenic (5 mg/kg) at E8 in C57BL6/J mice showed a significant increase in fetal body weight at E18 (Machado et al. 1999). Interestingly, in another study, the birth weight of C57BL6/J female pups exposed to 10 ppb arsenic from E8 to birth was not different from that of controls, and no differences in weight were observed when the pups reached 8 weeks of age (Ramsey et al. 2013b). Furthermore, the offspring of C57BL6/J mice exposed to 10 ppb arsenic throughout pregnancy exhibited no difference in body weight gain at weaning compared with controls (Kozul-Horvath et al. 2012). The different responses to exposure to 10 ppb arsenic *in utero* for CD-1 (our study) and C57BL6/J strains (Kozul-Horvath et al. 2012; Ramsey et al. 2013a) highlight the potential involvement of genetic background. Numerous studies have reported differences in sensitivity among different mouse strains for exposures to various chemicals (Bowen et al. 2010; Kimura et al. 2005; Robinson et al. 2010; Yan et al. 2011). It is generally accepted that C57BL6/J and related strains exhibit increased sensitivity to arsenic and cadmium based on observations of developmental malformations after exposure to these chemicals (Hovland et al. 1999; Machado et al. 1999; Ramsey et al. 2013a). Differences in the window and route of exposure as well as differences in genetic background could contribute to the variability of the outcomes following *in utero* arsenic exposure.

In addition to the incidence of obesity, glucose intolerance was observed in female mice exposed to 10 ppb and 45.5 ppm arsenic *in utero*. It was reported that adult mice exposed to ≥ 50 ppm arsenic developed impaired glucose tolerance (Hill et al. 2009; Paul et al. 2011). A recent study concluded that exposure of adult mice to 3 ppm sodium arsenite for 16 weeks resulted in altered glucose metabolism and pancreatic function (Liu et al. 2014). In human adults, chronic arsenic exposure (> 100 ppb) was associated with diabetes (Islam et al. 2012; Tseng et al. 2000). Type 2 diabetes was also correlated with low to moderate levels of arsenic exposure (Navas-Acien et al. 2008). Under our experimental conditions, the significantly higher body weights observed in the animals exposed to 10 ppb arsenic were maintained through 6 months of age (a similar trend was observed for the animals exposed to 42.5 ppm arsenic). A trend for higher circulating levels of leptin and insulin was also detected in the arsenic-exposed groups. We suspect that under our experimental conditions, *in utero* arsenic exposure could cause permanent alterations in lipid metabolism, leading to obesity phenotypes such as body fat increase and glucose intolerance (Cheng et al. 2011; Kozul-Horvath et al. 2012). Obesity is associated with leptin resistance (reviewed by Myers et al. 2010), and the trend for higher circulating levels of leptin in arsenic-exposed mice could be a potential mechanism that deserves further investigation.

Although our findings revealed perturbations of puberty onset, obesity, and glucose metabolism induced by *in utero* arsenic exposure, the mechanism of action underlying these changes remains unknown. Most effects of arsenic exposure in adults are attributed to the activation of gene pathways that increase reactive oxygen species and oxidative stress (Ghatak et al. 2011; Kitchin and Ahmad 2003; Lu et al. 2014). Changes in DNA methylation were reported in the lungs of adult C57BL6/J mice following exposure to 50 ppm arsenic for 90 days (Boellmann et al. 2010). Changes in methylation status in the livers of adult C57BL6/J mice were found after 5 months of exposure to 50 ppm arsenic in drinking water (Nohara et al. 2011). Methylation changes in human cord blood have also been associated with *in utero* arsenic exposure (Koestler et al. 2013). Given that the exposure window of our study was restricted to the second half of fetal life, the adult onset of perturbations could derive from epigenetic changes as a consequence of *in utero* arsenic exposure. These epigenetic changes may have effects at the cellular and/or systemic level that alter metabolism and hormone production.

## Conclusion

The most interesting aspect of our results is that *in utero* exposure to 10 ppb arsenic, the U.S. EPA–approved level for drinking water, caused similar or even more detrimental effects on body weight and age of vaginal opening than the tumor-inducing 42.5-ppm level. In CD-1 mice, arsenic appears to exert different cellular effects that depend upon the dose and the timing and length of exposure. Further studies are needed to elucidate the potential mechanisms of action of arsenic in a dose-dependent manner.

## Supplemental Material

(3.2 MB) PDF
