# Supplementary material for: Effects of in Utero Exposure to Arsenic during the Second Half of Gestation on Reproductive End Points and Metabolic Parameters in Female CD-1 Mice
Source: Environ Health Perspect. 2015 Aug 21;124(3):336–43. doi: 10.1289/ehp.1509703 (PMC4786990; doi:10.1289/ehp.1509703)
Supplement: (3.2 MB) PDF [file ehp.1509703.s001.acco.pdf]

Environ Health Perspect
DOI: 10.1289/ehp.1509703

Note to Readers: *EHP* strives to ensure that all journal content is accessible to all readers. However, some figures and Supplemental Material published in *EHP* articles may not conform to 508 standards due to the complexity of the information being presented. If you need assistance accessing journal content, please contact ehp508@niehs.nih.gov. Our staff will work with you to assess and meet your accessibility needs within 3 working days.

Supplemental Material

Effects of *in Utero* Exposure to Arsenic during the Second Half of Gestation on Reproductive End Points and Metabolic Parameters in Female CD-1 Mice

Karina F. Rodriguez, Erica K. Ungewitter, Yasmin Crespo-Mejias, Chang Liu, Barbara Nicol, Grace E. Kissling, and Humphrey Hung-Chang Yao

Table of Contents

Figure S1: Effects of *in utero* arsenic exposure on (A) maternal weight gains during gestation (represented as ratio between weight at birth and weight at the beginning of treatment; control n=7; 10 ppb n=7; 42.5 ppm n=7); (B) numbers of pups per litter (Control n=7; 10 ppb n=7; 42.5 ppm n=7) and (C) body weights of fetuses at E18 (Control n=46; 10 ppb n=30; 42.5 ppm n=38). All bars indicate mean ± SE and * indicates P< 0.05 compared to control.

Figure S2: Effects of *in utero* arsenic exposure on the ovarian morphology (H&E stained; bar represents 100 um) at 21 days, 28 days and 6 months of age.

Figure S3: Effects of *in utero* arsenic exposure on serum levels (Means ± SE) of (A) estradiol, (B) testosterone, (C) progesterone, and (D) DHEA at 6 months and 1 year of age.

Environ Health Perspect
DOI: 10.1289/ehp.1509703

Supplemental Figure S1

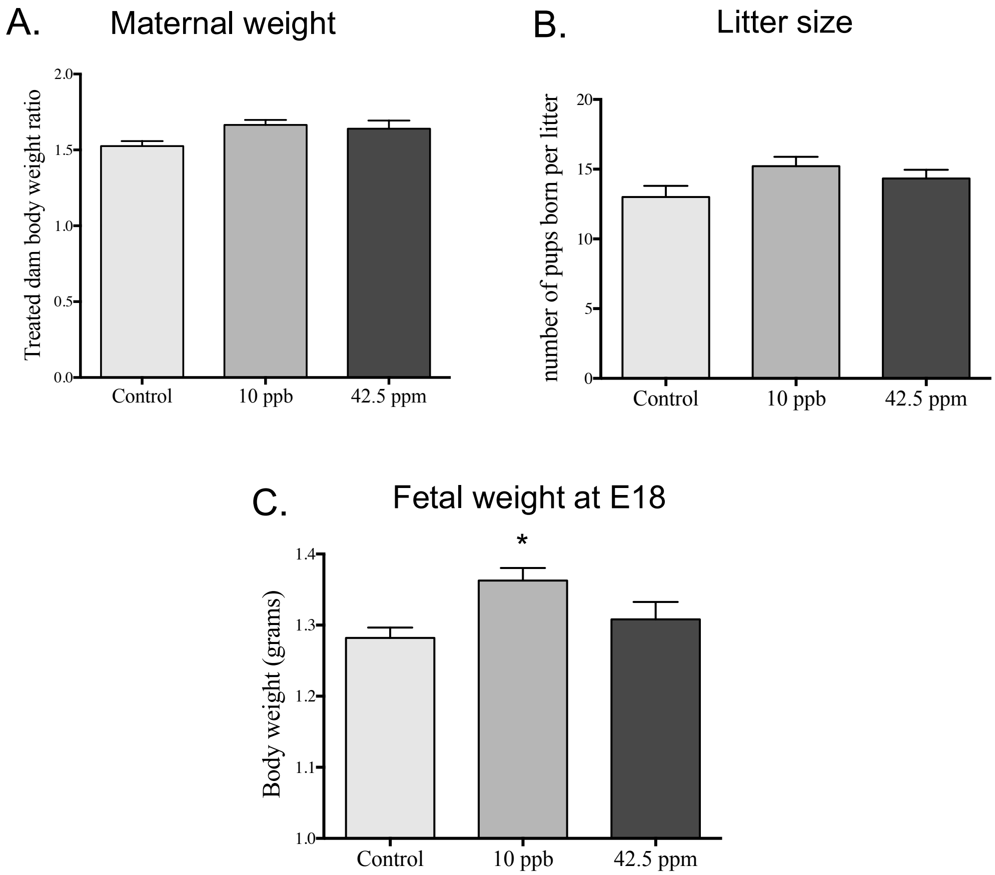

Figure S1: Effects of *in utero* arsenic exposure on (A) maternal weight gains during gestation (represented as ratio between weight at birth and weight at the beginning of treatment; control n=7; 10 ppb n=7; 42.5 ppm n=7); (B) numbers of pups per litter (Control n=7; 10 ppb n=7; 42.5 ppm n=7) and (C) body weights of fetuses at E18 (Control n=46; 10 ppb n=30; 42.5 ppm n=38). All bars indicate mean ± SE and * indicates P< 0.05 compared to control.

Environ Health Perspect
DOI: 10.1289/ehp.1509703

Supplemental Figure S2

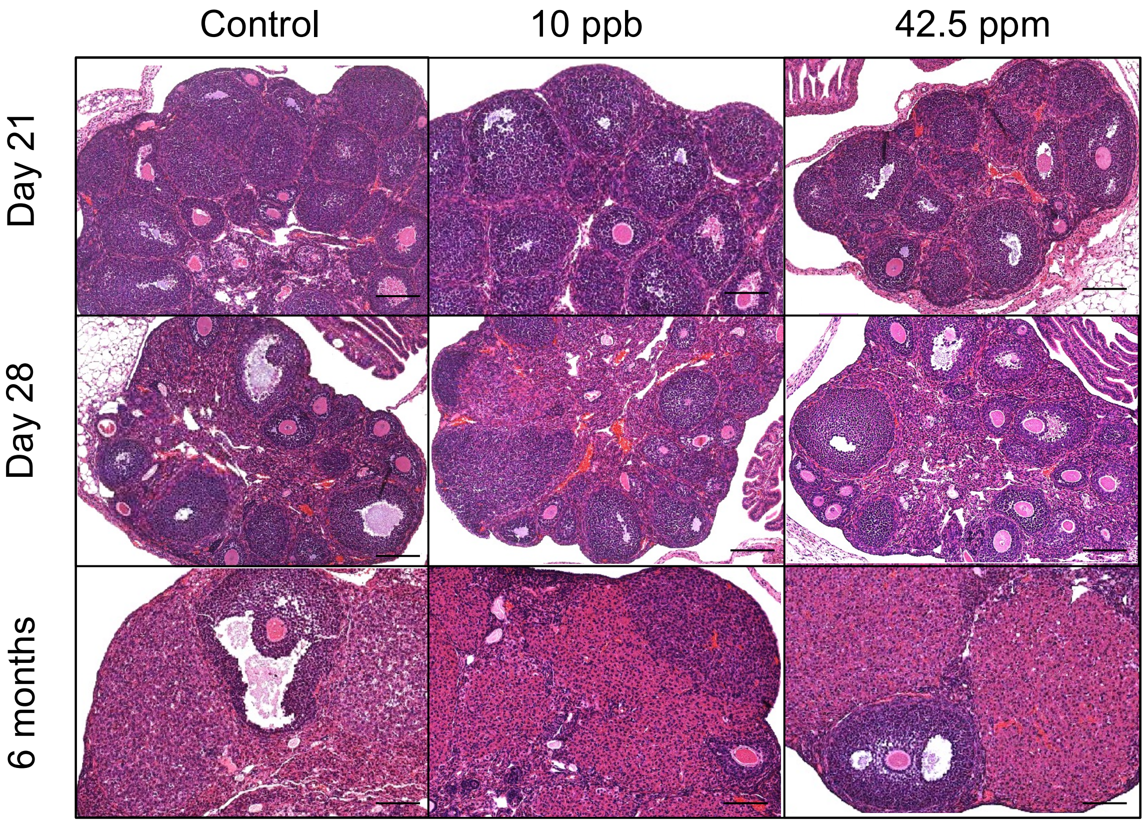

Figure S2: Effects of *in utero* arsenic exposure on the ovarian morphology (H&E stained; bar represents 100 um) at 21 days, 28 days and 6 months of age.

Environ Health Perspect
DOI: 10.1289/ehp.1509703

Supplemental Figure S3

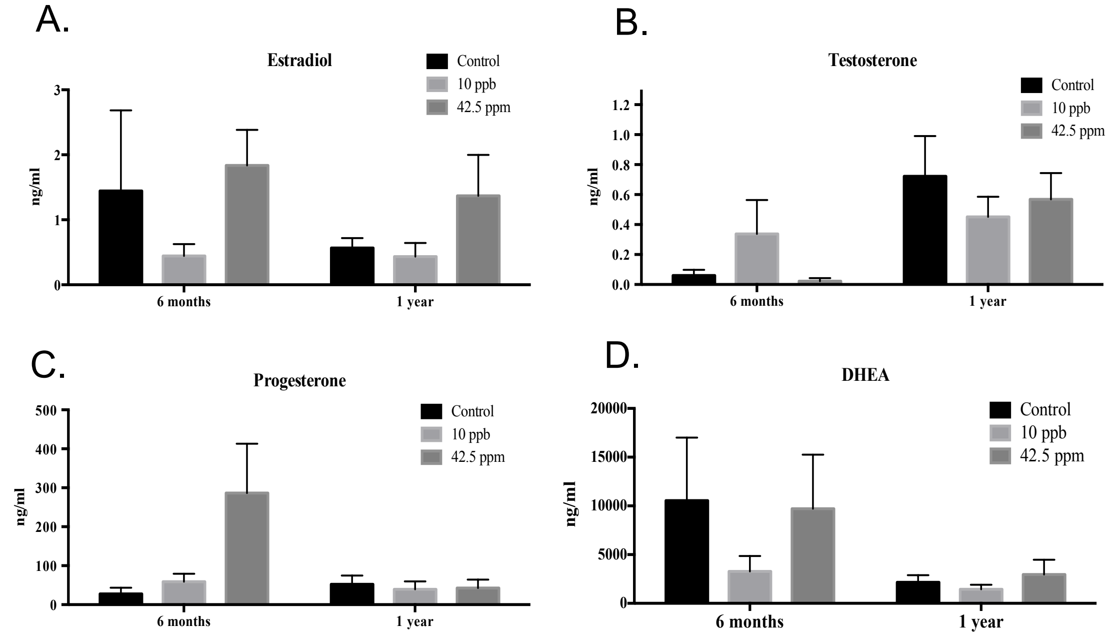

Figure S3: Effects of *in utero* arsenic exposure on serum levels (Means ± SE) of (A) estradiol, (B) testosterone, (C) progesterone, and (D) DHEA at 6 months and 1 year of age.
